# SuRFing the genomics wave: an R package for prioritising SNPs by functionality

**DOI:** 10.1186/s13073-014-0079-1

**Published:** 2014-10-14

**Authors:** Niamh M Ryan, Stewart W Morris, David J Porteous, Martin S Taylor, Kathryn L Evans

**Affiliations:** Centre for Genomic and Experimental Medicine, Institute of Genetics and Molecular Medicine, The University of Edinburgh, Western General Hospital, Crewe Road, Edinburgh, EH4 2XU UK; Centre for Cognitive Ageing and Cognitive Epidemiology, The University of Edinburgh, 7 George Square, Edinburgh, EH8 9JZ UK; MRC Human Genetics Unit, Institute of Genetics and Molecular Medicine, The University of Edinburgh, Western General Hospital, Crewe Road, Edinburgh, EH4 2XU UK

## Abstract

Identifying functional non-coding variants is one of the greatest unmet challenges in genetics. To help address this, we introduce an R package, SuRFR, which integrates functional annotation and prior biological knowledge to prioritise candidate functional variants. SuRFR is publicly available, modular, flexible, fast, and simple to use. We demonstrate that SuRFR performs with high sensitivity and specificity and provide a widely applicable and scalable benchmarking dataset for model training and validation.

Website: http://www.cgem.ed.ac.uk/resources/

**Electronic supplementary material:**

The online version of this article (doi:10.1186/s13073-014-0079-1) contains supplementary material, which is available to authorized users.

## Background

Linkage analyses and genome-wide association studies (GWASs) routinely identify genomic regions and variants associated with complex diseases [1]. Over 90% of disease-associated variants from GWASs fall within non-coding regions [2], underlining the importance of the regulatory genome in complex diseases. However, while there are a number of programs that identify putatively pathological coding variants, pinpointing the potential causal variants within non-coding regions is a major bottleneck, as the genomic signals that characterise functional regulatory variants are not fully defined and our understanding of regulatory architecture is incomplete [3].

Currently a number of large scale projects are under-way with the aim of genome-wide, systematic identification of functional elements, through a combination of biochemical assays followed by highly parallel sequencing [4]. This wealth of studies generating such data are typified by the Encyclopaedia of DNA Elements (ENCODE) project [5], Functional Annotation of the Mammalian Genome (FANTOM5) project [6], 1000 Genomes project [7] and the Epigenome Roadmap [8]. These endeavours provide genome annotation datasets for a range of genome marks, including histone acetylation and methylation [5], chromatin states [9], DNase hypersensitive sites (DNase HSs) [10,11], DNase footprints [12,13], transcription factor binding sites (TFBSs) [14,15], conserved sequences [16], enhancers [17] and polymorphisms [7]. These resources, which have been made publicly available via genome web browsers such as the UCSC Genome Browser [18] and the Ensembl Genome Browser [19], provide a rich dataset for functional predictions. Manual interrogation of genome browsers for a range of functional annotations simultaneously does not, however, scale well for large studies, lacks reproducibility, is unsystematic and is difficult to benchmark.

There is a need for a system that combines these annotation datasets, along with other genomic functional measures, to prioritise candidate variants for follow-up analyses. To address this need, we have developed the SuRFR tool (SNP Ranking by Function R package). SuRFR has the following advantages: simplicity; speed; modularity; flexibility; transparency (the output indicates which features have contributed to rankings); and ease of integration with other R packages.

In addition, we also introduce novel training and validation datasets that i) capture the regional heterogeneity of genomic annotation better than previously applied approaches, and ii) facilitate understanding of which annotations are most important for discriminating different classes of functionally relevant variants from background variants.

We show that SuRFR successfully prioritises known regulatory variants over background variants. Additional tests on novel data confirm the generalisability of the method. Moreover, we demonstrate that SuRFR either outperforms or performs at least as well as three recently introduced, approximately comparable, approaches [20-22] in the prioritisation of known disease variants from several independent datasets.

## Implementation

### Overview of SuRFR

SuRFR is based on the conceptually simple construct of a rank-of-ranks. Each variant is scored for its overlap with multiple types of annotation. For each annotation category, each variant is ranked from least likely to indicate function through to most likely. The ranks from multiple distinct annotation categories are averaged using a weighting model to produce a final, combined, weighted rank (R) of variant candidacy for the trait under consideration (Equation 1):

$$ R= ran{k}_i\left({\displaystyle \sum \Big({r}_{ij}.}{w}_j\right)\Big) $$

where *r*_*ij*_ is the rank of the *i*^*th*^ variant in the *j*^*th*^ annotation category, and *W*_*j*_ is the weight for the *j*^*th*^ annotation category.

Central to this approach is a weighting model that apportions the relative importance of each annotation type (a vector of multipliers, one for each annotation data source). The training and validation of weighting models is described in subsequent sections. SuRFR is distributed with three pre-trained weighting models that utilise publicly available data for variant annotation. The three pre-trained models are: a general model broadly applicable to any analysis (ALL); a model designed specifically for prioritising (rare) disease variants (DM); and a model for complex disease variants (DFP). Users are also free to specify their own weighting models. Some parameter types are additionally tunable within a pre-defined weighting model (for example, minor allele frequency (MAF)).

Training and validation of the weighting models were based on ten-fold cross-validation using a novel and broadly applicable data spiking strategy described in later sections.

SuRFR is implemented as an R package and is publicly available [23]. The input requirement is a tab-delimited text or bed file composed of chromosome number and start and end coordinates for each SNP (GRCh37/hg19 assembly); SuRFR builds a functional table based on these data. The SuRFR package can interact with a sister annotation package, 'SNP Annotation Information List R package' (SAILR). SAILR provides precompiled annotation tables for all variants from the 1000 Genomes project [7] for each of the four main populations (AFR, AMR, ASN, and EUR) from which users can extract a subset of SNPs of interest.

### Annotation sources

SuRFR incorporates information relating to a range of genomic and epigenomic annotation parameters known to correlate with regulatory elements and non-coding disease variants. Annotation data classes and sources are summarized in Additional file 1 and detailed below.

#### Minor allele frequency

MAFs were obtained from the 1000 Genomes EUR population for the cross-validation and model selection. The variants with the lowest MAF (rarest) were ranked highest. The optimal allele frequency range can, however, be tuned to suit any analysis.

#### Conservation

Genomic evolutionary rate profiling (GERP) estimates position-specific evolutionary rates and identifies candidate-constrained elements [24]. Constraint is measured in terms of rejection substitution (RS) scores, which are based on a comparison of the number of observed versus expected substitutions at each nucleotide position. SNP sites were ranked on the basis of their RS score. To prevent distortion of the rankings by positive selection and other confounding factors, we set all negative RS scores to zero prior to ranking.

#### DNase hypersensitivity

SNPs were ranked on normalised peak scores (maximum signal strength across any cell line) from genome-wide DNase HS data assayed in 125 cell types *(wgEncodeRegDnaseClusteredV2)* [25,26].

#### DNase footprints

This dataset comprised deep sequencing DNase footprinting data from the ENCODE project [27]. SNPs were ranked by the number of cell lines where DNase footprints were observed.

#### Chromatin states

We used chromatin states predicted by the combinatorial patterns of chromatin marks from the mapping of nine chromatin marks across nine cell lines [17]. Multivariable logistic regression on the full training/validation set was used to assess the relationship between chromatin states and variant type (regulatory or background variant) across the nine cell lines using the R function *glm*. Chromatin states were ranked from most to least informative; on the basis of β coefficients across the nine cell lines (Additional files 2 and 3). The highest ranking state from the nine cell lines was chosen to represent the chromatin state of each SNP.

#### Position

Ranking was determined by SNP position relative to gene features (exon, intron, splice site, promoter (defined as being within 1 kb of a transcription start site (TSS)), 10 kb upstream or downstream of a gene, intragenic, CpG islands, CpG shores). The ranking of the categories (Additional file 4) is based on enrichment data presented by Schork *et al*. [28] and Hindorff *et al*. [29]. UCSC gene annotation data ('Known Gene' gene predictions from sources such as RefSeq and GenBank) and the FANTOM5 CAGE data [16] were used to define TSSs.

#### Transcribed enhancers

Each SNP was assessed for overlap with CAGE-defined transcribed enhancers from the FANTOM5 project [30].

#### Transcription factor binding sites

TFBSs were identified from data based on ChiP-seq experiments for 161 transcription factors across 91 cell types and predicted transcription factor binding motifs from the ENCODE Factorbook repository (*wgEncodeRegTfbsClusteredV3*) [15,31]. The highest peak signal for any transcription factor across all cell lines was used to rank SNPs.

#### Annotation weightings

The SNP rankings for each of the annotation parameters were combined into a final rank-of-ranks by assigning weights to each parameter, thus adjusting their relative contribution to the final ranking of the SNPs. Different combinations of parameter weightings were assessed using cross-validation and a benchmarking dataset comprising non-coding disease and regulatory variants, and background genomic variants of unknown function (1000 Genomes variants located within the ENCODE pilot project regions).

### Construction of test datasets

For the purposes of model training and validation we constructed benchmark datasets (defined as being relevant, scalable, simple, accessible, independent and reusable [32]) by combining known functional variants with background datasets of variants. Several sources of both functional and background variants were used.

#### Non-coding variants with phenotypic impacts from the Human Gene Mutation Database

Non-protein-coding variants with reported phenotypic impacts were obtained from the Human Gene Mutation Database (HGMD Professional version, release December 2013), using the mutation table PROM, which contains substitutions that cause regulatory abnormalities [33]. Only variants of the subclasses ‘Disease causing mutation’ (DM), ‘Disease-associated polymorphism with additional supporting functional evidence’ (DFP) and ‘In vitro/laboratory or in vivo functional polymorphism’ (FP) were included.

The known variants were subdivided into three datasets by HGMD class: ALL (the full dataset of 1,827 variants with known disease effect or regulatory function); DM (644 variants of known disease causing mutations) only; and DFP (686 disease associated variants with functional evidence) only.

#### ENCODE background variants

To assess SuRFR’s ability to distinguish functional variants from non-functional, a control set of non-functional variants was required. However, training sets consisting of experimentally confirmed non-functional variants are still hard to come by and are limited in size. The ENCODE pilot project provides information on 44 regions across the genome that were selected around medically important genes and from regions with a cross-section of gene densities and non-exonic conservation scores [34]. Background variants were obtained by randomly sampling 170,892 SNPs located within the ENCODE pilot regions from the 1000 Genomes project EUR population [7].

#### Additional test datasets

For independent validation of SuRFR, we constructed annotation feature datasets for variant sets from a variety of sources. All of these contained variants with experimentally verified phenotypic impacts. Some of these datasets also contain background variants. All of these datasets were filtered to remove variants contained within the HGMD or ENCODE training and validation datasets.

##### Variants from the β-haemoglobin (*HBB*) locus

The HbVar database is a locus-specific database of human haemoglobin variants and thalassemias [35]. The HBB dataset constructed from HbVar data contains SNPs from the human haemoglobin beta gene, *HBB* (coding and non-coding), the true positive SNPs being variants that cause beta thalassemia (27 non-coding variants proximal to the *HBB* gene and 324 coding variants).

##### RAVEN regulatory variant dataset

To assess the ability of SuRFR to prioritise regulatory variants with no known disease association, we took advantage of a dataset designed to detect variants modifying transcriptional regulation [36], originally developed to train the web-based application RAVEN. The RAVEN true positive SNP set consists of 95 experimentally verified regulatory SNPs, and the control set, 3,856 background variants, all within 10 kb of genes with mouse homologs. An additional control set of background variants was constructed by randomly sampling the 1000 Genomes EUR dataset for SNPs that were matched for distance to the nearest TSS. This matched background set contains 9,500 variants.

##### ClinVar variant dataset

The ClinVar archive [37] provides a freely accessible collection of experimentally verified disease variants [38]. We compiled 128 variants (excluding mitochondrial variants) catalogued in the ClinVar archive (sourced from the GWAVA website [39]) into a known disease variant set. A background set of 150 variants classified as non-pathogenic was also taken from the GWAVA support website. An additional 58 non-exonic, non-coding SNPs were obtained directly from the ClinVar database and a background set of 5,800 1000 Genomes EUR SNPs matched for distance to the nearest TSS was generated for this dataset.

#### Complex trait related datasets

##### *SORT1* dataset

Musunuru *et al*. [40] investigated a chromosome 1p13 locus strongly associated with low-density lipoprotein cholesterol (LDL-C) levels and cardiovascular disease. Fine-mapping of the 1p13 locus, using SNPs genotyped from approximately 20,000 individuals of European descent [41], identified 22 variants in the minimal genomic region responsible for LDL-C association, of which the six SNPs with the highest association were clustered in a 6.1 kb non-coding region. Luciferase assays and electrophoretic shift assays were used to demonstrate that one of the six SNPs, rs12740374, creates a binding site for the transcription factor C/EBP and alters liver-specific expression of the *SORT1* gene. We constructed an annotation table for the 22 variants from this analysis.

##### *EGR2* dataset

The early growth response 2 (*EGR2*) gene is considered a good candidate for systemic lupus erythematosus susceptibility (SLE). Myouzen *et al*. [42] searched for functional coding variants within this locus by sequencing 96 individuals with SLE and found no candidate variants. A case-control association study for SLE of the 80 kb region around the *EGR2* gene identified a single SNP with a significant *P*-value. Functional characterisation (electrophoretic shift assay) of the SNPs in complete linkage disequilibrium (LD) with this tagging SNP showed that two SNPs had allelic differences in binding ability. Luciferase assays performed on these two SNPs showed that one (rs1412554) increased expression by 1.2-fold while the second (rs1509957) repressed transcriptional activity. The 35 proxy SNPs that are in perfect R-squared (R^2^ = 1.0) with the tagging SNP were annotated to test method performance.

##### *TCF7L2* dataset

In a search for variants associated with type 2 diabetes, (T2D) Gaulton *et al*. [43] identified known SNPs in strong LD with reported SNPs associated with T2D or fasting glycaemia. Of these variants, they identified six variants at the *TCF7L2* locus, one being a GWAS-significant SNP, rs7903146, and the other five being in LD with that tagging SNP). Using luciferase assays, they observed allelic differences in enhancer activity for the tagging SNP, rs7903146. These six SNPs defined a final dataset to assess SuRFR’s functionality.

### Cross-validation

Known functional and pathogenic variants were obtained from the HGMD database and split into three datasets: ALL (general class), DM (only disease mutations) and DFP (only disease-associated with further evidence of functionality). An equal number of background 1000 Genomes EUR variants from the ENCODE pilot regions were randomly selected. For the full (ALL) cross-validation analysis, known and background variants were split into a training/validation set (1,440 known and 1,440 background SNPs) and a hold-out test set (387 known SNPs and 169,452 background variants). The training/validation set was further randomly split into 10 folds for cross-validation.

A modified grid search algorithm, incorporating multivariable regression, was used for parameter optimisation. Multivariable regression performed on the full training/validation set was used to guide the parameter boundaries of the grid search algorithm (Additional file 5). Using brute force permutation of integer parameter values parameter weightings were permuted (n = 450,000) across the three models. Performance was measured using receiver operating characteristic (ROC) curves and area under the curve (AUC) statistics using the ROCR R package [44]). Maximum AUC with a threshold acceptable performance error <0.005 was the objective parameter optimised for weighting parameter selection.

Multiple very similar scoring models existed: the AUCs of the top 1% of weightings differed by less than 0.003 (Δ AUC ALL: 0.00258; Δ AUC DM: 0.00211; Δ AUC DFP: 0.00108), arguing for a smooth parameter space with few fine-grained local optima.

The 10-fold cross-validation was repeated for the HGMD subclasses DM (512 variants) and DFP (534 variants). The differences between the mean training AUCs and validation AUCs were used to calculate performance errors. Three models were developed from this analysis and incorporated in the R package: a general model, 'ALL'; a model specifically designed to identify (rare) disease mutations, 'DM'; and a model for complex disease variants (GWAS or common variants), 'DFP'. For each of the three dataset classes, the best model was run on the hold-out test dataset (similarly divided by variant class into ALL, DM and DFP categories). Generalisation errors were calculated by comparing test AUCs to the mean validation AUCs. Performance and generalisation errors were calculated to assess how consistently each model performed during cross-validation and to predict how well they would perform on novel data.

## Results and discussion

### Cross-validation analysis of genomic features using HGMD regulatory variants

Our goal was to design and test a method for the prioritisation of candidate functional SNPs. One of the greatest challenges faced in the development of a predictive method, such as this, is the need for systematic and impartial performance evaluation. Two critical factors in performance evaluation are i) good benchmarking datasets and ii) the use of appropriate statistical evaluation methods [32].

Non-coding variants with reported phenotypic impacts were obtained from HGMD. These variants were subdivided into three datasets: ALL (the full HGMD dataset, 1,827 SNPs); DM (known disease causing variants, 644 SNPs); and DFP (disease-associated variants with functional evidence, 686 SNPs). In each case, an equal number of background variants was obtained by randomly sampling SNPs from the 1000 Genomes project (EUR) that were located within the ENCODE pilot project regions. Although this background set will contain true functional variants, it has the advantage of providing insight into the impact different genomic backgrounds have on performance, making it an excellent benchmark dataset. In addition, a benchmark dataset should be relevant, accessible, reusable, representative of the data under investigation, composed of experimentally verified variants and applicable to the evaluation of other tools. The combination of phenotypically functional variants from HGMD and ENCODE pilot region background variants fulfils all of these criteria.

We used 10-fold cross-validation to assess the performance and gerenalisability of SuRFR on the three datasets. All three datasets were divided into training, validation and hold-out test subsets. For each dataset, each of the three subsets comprised non-overlapping sets of SNPs. This was an important consideration as it prevented over-fitting of the derived models.

**Figure 1:**
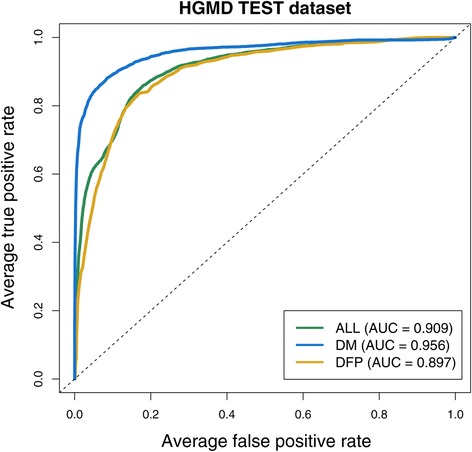
**ROC curves for the three SuRFR models on the hold-out test datasets.** Mean ROC curves and AUCs for the top three SuRFR models from the cross-validation, run on the hold-out test dataset. True positive rate (x-axis) plotted against false positive rate (y-axis) for each of the three models: ALL (green line); DM (blue line); and DFP (golden line). The dotted grey line indicates random chance.

We assessed SuRFR’s performance via ROC curve and AUC statistics. Optimum parameters were chosen for each model on the basis of average training/validation AUCs and corresponding error rates (see Implementation section). The AUCs obtained for each model when run on the training, validation and, crucially, the hold-out test sets were high (from 0.90 to 0.98), indicating that each model successfully prioritises known regulatory variants over background variants (Table 1, Figure 1). Moreover, the performance and gerenalisation errors were low (<0.035), indicating that the method would be likely to perform equally well on novel data.

**Table 1:** **Average training, validation and test AUCs for the three SuRFR models run on the cross-validation datasets**

**Model**	**Training AUC**	**Validation AUC**	**Test AUC**	**Performance error**	**Gerenalisation error**
ALL	0.944	0.944	0.909	0.000	0.035
DM	0.976	0.976	0.956	0.000	0.020
DFP	0.912	0.908	0.897	0.004	0.013

The AUCs and error rates from cross-validation for the three SuRFR models. Column 1 shows the three models (ALL, DM, DFP). Columns 2 and 3 show the average training AUCs and validation AUCs, respectively, for each of the three models from the 10-fold cross-validation analysis. The performance error (column 5) shows that the difference between the training and validation AUCs is small. Column 4 shows the average test AUCs achieved by each of the three models run on the hold-out datasets. The low gerenalisation errors in column 6 and the AUCs from the test datasets show that SuRFR is likely to gerenalise and perform equally well on novel data.

### Different sets of genomic features characterise different classes of regulatory variants

The 10-fold cross-validation and subsequent testing on the hold-out dataset showed that each class of functional variant was best prioritised by different combinations of genomic annotations (Table 2). DM variants were consistently ranked higher than background variants by a large range of annotation models. In the main, the DM variants give rise to high penetrance and Mendelian disorders, that is, disorders with more severe phenotypic outcomes. Such variants could, perhaps, be expected to result in substantial functional changes. As a group, these variants would be likely to be associated with changes across many functional annotation categories, thus they can be identified by a range of functional annotation weightings. In contrast, the DFP variants are likely to result in more subtle changes to function and, we could hypothesise, would be more difficult to detect. In fact, the DFP variants required a very specific combination of annotation weightings, combining position, chromatin states, DNase footprints, enhancers and TFBSs, with conservation having no impact.

**Table 2:** **Details of the weighting models for each of the three variant classes**

**Model**	**MAF**	**Conservation**	**Chromatin states**	**DNase HS**	**Position**	**DNase footprints**	**Enhancers**	**TFBSs**
ALL	0	1	1	0	8	0	1	3
DM	12	2	6	1	15	1	0	5
DFP	0	0	3	1	15	3	5	2

The first column lists the three weighting models (ALL, DM and DFP). Each subsequent column represents a different annotation class. The values represent the weightings of each annotation class defined in each weighting model.

Overall, we found SNP position to be the most informative feature with respect to functionality for all three classes of functional variants. This finding is consistent with evidence in the literature, which shows that a regulatory site’s influence on expression falls off almost linearly with distance from the TSS in a 10 kb range [45], and that disease variants are enriched in certain genomic positions, including coding regions and promoters, over intronic and intergenic regions [28].

The ranking of the different classes of chromatin states were chosen based on multivariable regression of the full training and validation dataset (Additional file 2), the promoter and strong enhancer chromatin states ranking above the other classes. Chromatin states were also found to be good markers of functionality across all variant classes. This finding is in keeping with the literature: for example, disease variants are over-represented in genomic regions characterised by particular chromatin states, such as strong enhancers [17], more often than others. As we prioritise SNPs in strong enhancers above most other chromatin states, our results are in keeping with these findings.

TFBSs played a role in the ranking of all three classes of regulatory variants. This is not unexpected, as changes to TFBSs may alter transcription factor binding and thus have an impact on function.

Non-coding disease-associated GWAS variants are concentrated in DNase HSs, and thus putative regulatory sites [2]. It is unsurprising, therefore, that we find that DNase HSs and footprints are important markers of functionality. Our analysis shows that DNase HS clusters and DNase footprints are highly correlated, making it difficult to separate any individual effects. In our analysis, DNase footprints on their own provide as much information as using the two features together. This is likely to be because they provide overlapping information: DNase HSs mark regions of open chromatin and DNase footprints mark the (more specific) regions of transcription factor occupancy.

Ranking SNPs on MAF (low frequency scoring highest) was very effective in the prioritisation of DM class variants over background variants, but was not important in the ALL or DFP classes. This is likely to be due to the fact that DM variants are most likely to be Mendelian or highly penetrant, making them more likely to be rare, whereas the DFP class tend to be those associated with lower penetrance, complex traits and are, therefore, more likely to have higher MAFs.

We found that conservation is not a particularly informative annotation, playing a minor role in the identification of DM variants, making an even smaller contribution to identifying ALL variants, and not contributing at all to the identification of DFP variants. There are a number of methods used to assess variant function that solely rely on conservation (Table two from Cooper & Shendure, [4]) and others have shown that conservation can be used to discriminate functional regulatory variants from background variants [20]. However, our finding supports those studies that have shown that conservation is a poor predictor of regulatory function [46] and is consistent with findings of extensive regulatory gain and loss between lineages, indicating that there is variation in regulatory element positions across evolution [47].

Transcribed enhancers do not correlate with the DM class and only modestly with the ALL class of regulatory variants but do provide information on functionality for the DFP variants, leading to the hypothesis that the DFP variants are more likely to be of weak effect or have tissue-specific roles compared with the other classes. It is difficult to judge the significance of this due to the current, relatively small dataset.

### Performance of SuRFR on additional test datasets

To further test the generalisability of our method we tested our ALL, DM, and DFP models on two additional test datasets. The first comprises variants from the *HBB* locus (HBB dataset) [35] that are known to cause thalassemia, which allow assessment of SuRFR’s ability to prioritise regulatory Mendelian disease variants; the second was the RAVEN dataset, which consists of experimentally validated non-coding regulatory variants with no known disease-association, located within 10 kb of genes with mouse homologs [36].

**Figure 2:**
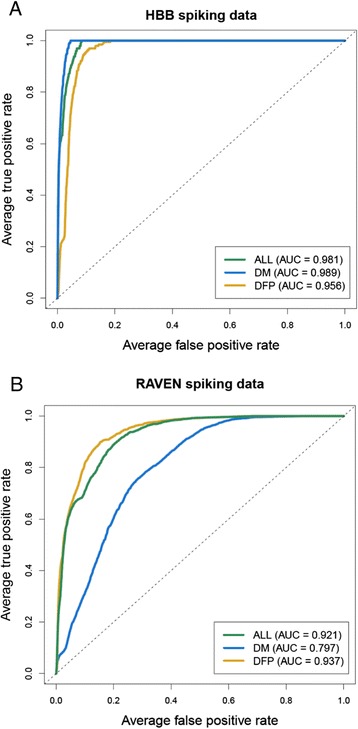
**ROC curves for the three SuRFR models run on the HBB and RAVEN datasets. (A)** HBB analysis; **(B)** RAVEN analysis. Mean ROC curves (true positive rate (x-axis) plotted against false positive rate (y-axis)) and AUCs for the three SuRFR models (ALL, green; DM, blue; DFP, gold) run on the HBB non-coding **(A)** and RAVEN non-coding **(B)** regulatory variants spiked into the ENCODE background datasets. The dotted grey line indicates random chance.

As the HBB dataset does not contain background variants, the 27 non-coding HBB variants were spiked into the 44 ENCODE regions and the average AUC across the regions calculated. All three models performed extremely well on this dataset, with AUCs ranging from 0.95 to 0.98 (Figure 2A), confirming the ability of SuRFR to correctly prioritise pathogenic variants with high accuracy.

The 95 non-coding RAVEN variants were also spiked into the ENCODE regions, as for the HBB variants, and the performance of SuRFR was ascertained (Figure 2B). On this dataset, both the general (ALL) and DFP models performed well (AUCs of 0.95 and 0.94, respectively); however, the DM model’s AUC was 0.80. This result was not unexpected as the RAVEN variants comprise known regulatory, rather than disease-causing, variants, and as such would not be expected to be discriminated by the disease variant model.

**Figure 3:**
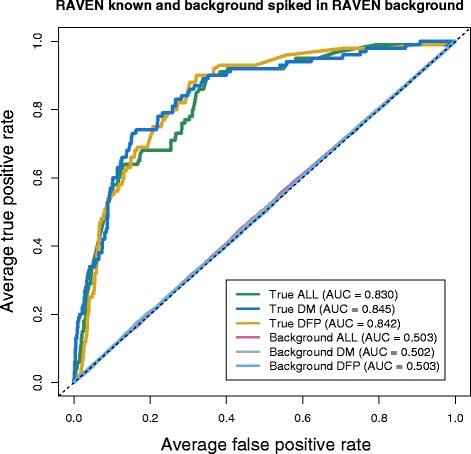
**Performance of SuRFR on regulatory versus background variants.** ROC curves and AUCs for the three models of SuRFR run on true RAVEN variants (experimentally verified) or negative RAVEN variants (background variants set as 'true'). The three 'True' analyses (ALL, green line; DM, dark blue line; DFP, golden line) all perform consistently well, with an average AUC of 0.845, whereas the 'Background' as true analyses showed SuRFR does not detect background variants any more than you would expect by chance (the grey dotted line).

The RAVEN dataset additionally contains 3,856 background variants, matched by position to the experimentally verified regulatory variants. The RAVEN regulatory variants were compared against the background variants and produced AUCs of 0.851, 0.839 and 0.844 for the ALL, DM and DFP models, respectively (Figure 3).

### Background variants as known functional variants

As a negative control, we also tested SuRFR’s ability to prioritise a subset of background variants versus other background variants. The RAVEN background variant set consists of 3,856 variants that are within the 10 kb region upstream of genes that are conserved between mice and humans. One-hundred randomly sampled subsets of 95 variants from this RAVEN background dataset were redefined as 'known' and spiked into the RAVEN background dataset. The average AUC calculated across these 100 sets was 0.50, indicating background variants are not prioritised any better than would be expected by chance (Figure 3). In contrast, the 95 RAVEN background variants spiked into the same background set (see above), achieved AUCs ranging from 0.84 to 0.85, demonstrating the ability of the method to prioritise functional variants better than non-functional variants.

### Comparison with alternative methods

We compared SuRFR’s ability to prioritise known pathogenic variants against three additional tools that prioritise non-coding variants using a somewhat comparable approach: GWAVA, CADD and FunSeq. GWAVA uses a modified random forest algorithm, written in the Python language, to prioritise non-coding variants [20]. CADD provides a single measure (C score) that has been pre-computed for the entire genome. C scores are based on integration of multiple annotations [21]. FunSeq is a method for prioritising cancer drivers. Prioritisation is based upon the assessment of patterns of multiple functional annotations. The authors state that FunSeq will be most effective in the analysis of tumour genomes, but can also be applied for the analysis of germ line mutations [22].

**Figure 4:**
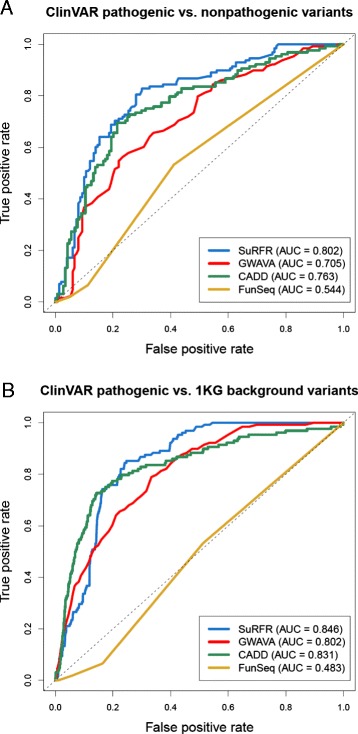
**Comparison of SuRFR, GWAVA, CADD and FunSeq on two ClinVar datasets. (A,B)** ROC curves (true positive rate versus false positive rate) and AUCs for SuRFR, GWAVA, CADD and FunSeq run on ClinVar pathogenic versus non-pathogenic variants **(A)** and ClinVar pathogenic versus matched 1000 Genomes background variants **(B)**. SuRFR outperforms all three methods on both of these datasets, with AUCs of 0.802 and 0.846 versus 0.705 and 0.802 for GWAVA, 0.763 and 0.831 for CADD and 0.544 and 0.483 for FunSeq on the two datasets, respectively.

To compare SuRFR with these methods, we used an independent dataset of 128 pathogenic variants from the ClinVar archive of disease variants (see Implementation section). This dataset excludes mitochondrial variants, as SuRFR has been trained on nuclear, not mitochondrial, variants and relies heavily on functional data that are not applicable to mitochondrial variants (most notably, histone modifications and DNase HS data). These were compared against two background sets: a background set of 150 'non-pathogenic' ClinVar variants and 19,400 variants identified as part of the 1000 Genomes project [7], selected by Ritchie *et al*. [20] for their assessment of GWAVA’s performance, which were matched with the pathogenic variants for distance to the nearest TSS. None of the three datasets contained variants used to train SuRFR, GWAVA, CADD or FunSeq, allowing rigorous comparison of the methods’ performances. SuRFR was run using the DM model, as it is the most appropriate model for this data type. GWAVA was similarly run using the TSS model, as this was used by Ritchie *et al*. in their original analysis [20]. CADD has no alterable parameters; however, FunSeq was run using the ‘personal genome’ option and a MAF cutoff of 0.1. SuRFR was able to discriminate the pathogenic variants from background variants with AUCs of 0.80 and 0.85, respectively, while on the same datasets the AUCs were 0.71 and 0.80 for GWAVA, 0.76 and 0.831 for CADD, and 0.544 and 0.483 for FunSeq (Figure 4A,B).

To test the performance of each method on a purely non-exonic, non-coding clinical dataset (as the ClinVar data used in the GWAVA paper include synonymous, non-synonymous and UTR exonic variants), we extracted 58 such variants directly from the ClinVar database and generated a background set matched by distance to the nearest TSS, 100 times the size of the true positive set. Interestingly, all of the tools performed similarly on this dataset: the AUC for SuRFR (DM model) was 0.671, 0.629 for GWAVA (TSS model) and 0.692 for CADD (Additional file 6). None of them were as good at identifying this non-exonic, non-coding dataset against this stringently matched background set compared with the other ClinVar datasets. On this dataset GWAVA would have an advantage over the other tools, as the TSS model was specifically trained on this type of data. The lower AUC for SuRFR, however, is likely to represent the lowest estimate of SuRFR’s performance, as by comparing regulatory variants with control variants matched by distance to TSS, we are effectively removing position from consideration. While we have shown that position is the most important feature in SuRFR’s variant prioritisation, we can assume SuRFR’s ability to distinguish pathogenic from matched background variants is due to the additional features included in our model. As position matching of background variants is an unrealistically harsh testing environment, we expect and observe better performance in real world scenarios (Table 3).

**Table 3:** **Rankings of experimentally validated regulatory variants from three real world analyses for SuRFR, GWAVA and CADD**

	**Total number of variants**	**SuRFR ranking of functional variant**	**GWAVA ranking of functional variant**	**CADD ranking of functional variant**
SORT1	22	1st out of 22	6th out of 22	20th out of 22
EGR2	35	1st out of 35	2nd out of 35	18th out of 35
TCF7L2	6	2nd out of 6	2nd out of 6	2nd out of 6

SuRFR performs consistently well against GWAVA and CADD on these three datasets.

SuRFR and GWAVA were also tested on a set of coding disease variants for β thalassemia located within the *HBB* gene. Although neither method is specifically designed to prioritise coding variants, both were extremely good at discriminating the coding variants from the ENCODE background sets (Additional file 7), SuRFR and GWAVA achieving AUCs of 0.998 and 0.975, respectively. As the HBB and RAVEN non-coding variants overlapped with variants used in the GWAVA training and validation datasets, it was not possible to compare SuRFR and GWAVA’s performance on these data. It was, however, possible to compare SuRFR’s performance with CADD's on this dataset. For this study, we combined the RAVEN experimentally verified regulatory variants with 9,500 background variants, matched by distance to the nearest TSS (100 control variants for each true positive variant). The AUC for SuRFR on this dataset was 0.702, while CADD achieved a more modest performance, with an AUC of 0.608 (Additional file 8).

To establish next how well SuRFR performs compared with GWAVA and CADD on variants related to complex traits, we ran all three methods on three published analyses identifying regulatory variants associated with disease risk (see Implementation section).

#### SORT1: analysis of a chr1p13 locus associated with low-density lipoprotein levels and cardiovascular disease

Musunuru *et al*. [40] showed that a region of chromosome 1p13 was associated with LDL-C. They carried out functional analysis on 22 variants from the locus and identified rs12740374 as the most likely functional candidate. We ran the 22 candidate variants through SuRFR and compared their ranking with GWAVA and CADD’s rankings [20]. SuRFR successfully ranked rs12740374 1st out of the 22, whereas GWAVA ranked it 6th out of 22 and CADD ranked it 20th out of 22 (Table 3).

#### *EGR2: Evaluation of variants from the* EGR2 *locus associated with systemic lupus erythematosus*

The 80 kb chr10q21 candidate locus for SLE contains a total of 237 variants with a MAF >0.10 from the 1000 Genomes ASN population [7]. When all 237 SNPs were assessed by GWAVA, CADD and SuRFR, no tool was able to identify rs1509957 (a SNP found by Myouzen *et al*. [42] to have reduced activity in a reporter assay) within the top 10%. However, when only the 35 proxy SNPs in LD with the most significantly associated SNP from their association study for SLE were ranked, SuRFR ranked rs1509957 1st out of 35, GWAVA ranked it 2nd, and CADD ranked it 18th, highlighting the importance of using additional prior biological information to pre-filter variants to improve predictive power (Table 3).

#### *Study of type 2 diabetes-associated variants at the* TCF7L2 *locus*

Of the six variants within the T2D GWAS associated region at the *TCF7L2* locus, only one SNP showed significantly increased enhancer activity. GWAVA, CADD and SuRFR all ranked this variant second out of six (Table 3).

We have shown that SuRFR either outperforms or performs as well as GWAVA, and that both GWAVA and SuRFR substantially outperform CADD on the datasets tested here. The 'black box' nature of GWAVA’s design means that we are unable to comment on the reasons for the difference in performance between the two methods. However, Ritchie *et al*. [20] report that G + C content, conservation, DNase HSs, distance to the nearest TSS and some histone modifications contribute most to the discriminative power of GWAVA. While there are overlaps between the annotations used by the two methods there are also differences, and it seems likely that these differences contribute to the difference in performance between SuRFR and GWAVA. The training and validation approaches also differ and we would argue that our tripartite training, validation and testing splits of the initial data are better suited to avoid over-fitting than the GWAVA bipartite training and validation approach.

CADD was developed using an entirely different protocol involving a support vector machine trained to differentiate high-frequency human-derived alleles from an equal number (14.7 million) of simulated variants [21]. A wide range of annotations were assessed and combined into a single measure (C score) for each variant, which can be viewed as an estimate of deleteriousness. SuRFR either matches or outperforms CADD on all of the datasets we have tested. This may be because CADD is trained to differentiate high-frequency alleles from simulated variants of equal frequencies, whereas the datasets under test often contain a range of allele frequencies.

### Advantages of using SuRFR

Implementation of SuRFR in R has many advantages, including ease of use and of data management. In addition, code run times are short and the R environment provides a high level of flexibility. For example, the use of R facilitates incorporation of additional modules, functions and annotation data in the future; and integration with other R packages. This is a clear advantage over web-based methods, where there may be issues of data security, control over parameter settings or flexibility to modify the underlying code. At every point during the running of the R package, users can understand the extent to which the various annotations contribute to the variant rankings, allowing construction of hypotheses based on the data obtained. This is a major advantage over 'black box' approaches such as GWAVA, where the user is unaware of the factors affecting variant rankings.

### Intended use of the software

This R package is intended to be used as an aid for genomics studies. We must, however, emphasise that SuRFR is predictive and does not take the place of experimental validation. Instead, it should be used as a guide to prioritising candidate variants to take forward for follow-up analysis.

### Limitations

SuRFR is not currently designed to discriminate between coding variants of differing impact; however, many existing software packages perform this task well [4].

SuRFR, and any other comparable method, is likely to discriminate against long-range enhancers, due to the strong influence of SNP position (score increasing with proximity to genes). This is currently a difficult issue to address, as known variants are biased towards coding and promoter variants and no relevant datasets exist to train methods in the discrimination of true long-range enhancers.

All methods tested here performed less well on matched non-exonic, non-coding variants (Additional file 6). Again, this is likely to be due to a lack of knowledge, sufficiently comprehensive genomic measures and appropriate training datasets.

### Outline of planned future development

There is scope for extending SuRFR. Planned future developments under consideration include expanding the collection of annotations to assess the impact of coding variants and investigation of additional annotations that may correlate with regulatory elements (for instance, expression quantitative trait loci data). In addition, we would hope to improve SuRFR’s flexibility by i) linking it in with other R packages (for example, next-generation sequencing packages and methylation and expression analysis packages), and ii) provide additional utility for user customisation.

We would also like to extend SuRFR's remit to assess indels. This goal is currently somewhat hampered by a relative dearth of appropriate training and validation data.

## Conclusions

Assessing the impact of non-coding variants is currently a major challenge in complex trait genetics. We have developed a system that combines a unique collection of data from multiple functional annotation categories to prioritise variants by predicted function. The modular design and tunable parameterisation of SuRFR allows for the simple and efficient incorporation of publicly available data and prior biological knowledge into the ranking scheme.

The R package provides three models: a general model for any analysis (ALL); a model designed specifically for prioritising (rare) disease variants (DM); and a model for complex disease variants (DFP). Alternatively, SuRFR allows users to specify their own custom model. This method has been tested on known regulatory and disease variants and a proposed benchmark background variant dataset and has been shown to perform with high sensitivity and specificity. SuRFR also has the ability to prioritise coding and non-coding functional variants.

Our analysis has provided insight into the extent to which different classes of functional annotation are most useful for the identification of known regulatory variants. We have also shown that SuRFR either outperforms, or performs at least as well as, comparable SNP prioritisation approaches, whilst benefiting from the advantages that come from being part of the R environment.

## Availability and requirements

**Project name:** SuRFR

**Project home page:**http://www.cgem.ed.ac.uk/resources/

**Operating system(s):** unix/linux

**Programming language:** R

**Other requirements:** bedtools and tabix

**License:** Artistic-2.0

**Any restrictions to use by non-academics:** No

## Additional files

Additional file 1: Table S1. Annotation data and sources. Additional file 2: Table S2. Chromatin state multivariable regression β coefficients. Additional file 3: Table S3. Chromatin state rankings. Additional file 4: Table S4. Position category rankings. Additional file 5: Table S5. Grid search parameter boundaries. Additional file 6: Figure S1. Comparison of SuRFR, GWAVA and CADD on an additional, non-coding ClinVar dataset. ROC curves (true positive rate versus false positive rate) and AUCs for SuRFR, GWAVA and CADD run on a non-exonic, non-coding dataset of ClinVar pathogenic variants versus a matched 1000 Genomes background variant set. SuRFR, GWAVA and CADD perform to a similar level on these data. Additional file 7: Figure S2. ROC curves and AUCs of SuRFR versus GWAVA on *HBB* coding variants. The plot shows the performance of SuRFR and GWAVA in terms of true positive rates (x-axis) and false positive rates (y-axis), plotting ROC curves (SuRFR, blue; GWAVA, red) against performance expected by chance (grey dotted line). This figure shows that both methods are very good at prioritising functional coding variants over background variants. Additional file 8: Figure S3. ROC curves and AUCs for SuRFR versus CADD on RAVEN regulatory variants versus a matched control set. The plot shows the performance of SuRFR and CADD in terms of true positive rates (x-axis) and false positive rates (y-axis), plotting ROC curves (SuRFR, blue; CADD, green) against performance expected by chance (grey dotted line). This figure shows that both methods prioritise functional regulatory variants over matched background variants to a similar extent.

## Abbreviations

AUC: area under the ROC curve
DNase HS: DNase hypersensitive site
GWAS: genome-wide association study
HGMD: Human Gene Mutation Database
LD: linkage disequilibrium
LDL-C: low-density lipoprotein cholesterol
MAF: minor allele frequency
ROC: receiver operating characteristic curve
RS: rejection substitution
SLE: systemic lupus erythematosus
SNP: single-nucleotide polymorphism
T2D: type 2 diabetes
TFBS: transcription factor binding site
TSS: transcription start site
UTR: untranslated region
