# Supplementary material for: SuRFing the genomics wave: an R package for prioritising SNPs by functionality
Source: Genome Med. 2014 Oct 14;6(10):79. doi: 10.1186/s13073-014-0079-1 (PMC4224693; doi:10.1186/s13073-014-0079-1)
Supplement: Additional file 1: Table S1. — Annotation data and sources. [file 13073_2014_79_MOESM1_ESM.doc]

**Additional file Table S1. Annotation data and sources**

| Annotation | Details | Source | Download date |
| --- | --- | --- | --- |
| Minor Allele Frequency (MAF) | 1000 Genomes phase-1 EUR population | ftp://ftp.ensembl.org/pub/release-72/variation/vcf/homo_sapiens/ | Jun-14 |
| RS numbers | 1000 Genomes phase-1 EUR population | ftp://ftp.ensembl.org/pub/release-72/variation/vcf/homo_sapiens/ | Jun-14 |
| Genomic Evolutionary Rate Profiling (GERP) scores | UCSC hg19 release, hg19.GERP.bed | MySQL --user=genome --host=genome-mysql.cse.ucsc.edu -A -D hg19 -P 3306 | Jul-13 |
| Chromatin states | UCSC hg19 release, wgEncodeBroadHmm* x nine cell lines | MySQL --user=genome --host=genome-mysql.cse.ucsc.edu -A -D hg19 -P 3306 | Apr-13 |
| DNase hypersensitivity sites | UCSC hg19 release, wgEncodeRegDnaseClustered | MySQL --user=genome --host=genome-mysql.cse.ucsc.edu -A -D hg19 -P 3306 | Mar-14 |
| DNase footprints | ENCODE DNase footprints | ftp://ftp.sanger.ac.uk/pub/resources/software/gwava/v1.0/source_data/encode/ | Mar-14 |
| Transcription Factor Binding Sites | wgEncodeRegTfbsClusteredInputsV3 | MySQL --user=genome --host=genome-mysql.cse.ucsc.edu -A -D hg19 -P 3306 | Mar-14 |
| Gene names, exons, introns, splice sites | UCSC hg19 release, knownGene | MySQL --user=genome --host=genome-mysql.cse.ucsc.edu -A -D hg19 -P 3306 | Apr-13 |
| CpG islands | UCSC hg19 release, cpgIslandExt | MySQL --user=genome --host=genome-mysql.cse.ucsc.edu -A -D hg19 -P 3306 | Apr-13 |
| FANTOM5 CAGE data | FANTOM5 robust transcription start sites | http://fantom.gsc.riken.jp/5/datafiles/phase1.0/ | Mar-14 |
| Transcribed Enhancers | Transcribed Enhancer Atlas, permissive enhancer set | http://enhancer.binf.ku.dk/Pre-defined_tracks.html | Mar-14 |

Table showing the annotation classes defined in SuRFR, the data used for each annotation category, their sources and the date they were downloaded.
