# Supplementary material for: SuRFing the genomics wave: an R package for prioritising SNPs by functionality
Source: Genome Med. 2014 Oct 14;6(10):79. doi: 10.1186/s13073-014-0079-1 (PMC4224693; doi:10.1186/s13073-014-0079-1)
Supplement: Additional file 2: Table S2. — Chromatin state multivariable regression β coefficients. [file 13073_2014_79_MOESM2_ESM.doc]

**Additional file Table S2: Chromatin state multivariable regression β coefficients**

| chromatin state | Average β coefficient across nine cell lines | Std. Error | Pr(>|z|) |
| --- | --- | --- | --- |
| 15_Repetitive/CNV | 13.399 | 322.129 | 0.966 |
| 1_Active_Promoter | 3.551 | 0.353 | 0.000 |
| 2_Weak_Promoter | 2.127 | 0.338 | 0.000 |
| 3_Poised_Promoter | 2.565 | 0.558 | 0.006 |
| 6_Weak_Enhancer | 1.099 | 0.278 | 0.008 |
| 4_Strong_Enhancer | 1.500 | 0.389 | 0.025 |
| 12_Repressed | 0.404 | 0.142 | 0.029 |
| 9_Txn_Transition | -0.051 | 0.401 | 0.729 |
| 8_Insulator | 0.030 | 0.412 | 0.585 |
| 10_Txn_Elongation | -0.363 | 0.191 | 0.140 |
| 7_Weak_Enhancer | -0.072 | 0.244 | 0.541 |
| 11_Weak_Txn | -0.532 | 0.110 | 0.000 |
| 13_Heterochrom/lo | -0.659 | 0.057 | 0.000 |
| 5_Strong_Enhancer | 0.010 | 0.410 | 0.280 |
| 14_Repetitive/CNV | -4.811 | 246.088 | 0.867 |

Multivariable logistic regression for the 15 Ernst chromatin states run on the full training and validation dataset. Column 1 shows the 15 chromatin states as defined by Ernst et al . The average β coefficients across the nine cell lines (column 2) showed pronounced grouping of “like” categories with similar β coefficients. To simplify, we collapsed these similar categories into 10 classes of chromatin state. Taking the average rank produced a combined rank shown in Supplementary Table 3. The two ‘Repetitive/CNV’ categories were treated differently due to high standard error rates (column 3) and noise in the data. This class was positioned in the middle of the ranking, between classes that correlated positively with our data classes and those that had a negative correlation.
