# Supplementary material for: SuRFing the genomics wave: an R package for prioritising SNPs by functionality
Source: Genome Med. 2014 Oct 14;6(10):79. doi: 10.1186/s13073-014-0079-1 (PMC4224693; doi:10.1186/s13073-014-0079-1)
Supplement: Additional file 3: Table S3. — Chromatin state rankings. [file 13073_2014_79_MOESM3_ESM.doc]

**Additional file Table S3: Chromatin state rankings**

| chromatin state classes | Rank |
| --- | --- |
| Promoter | 10 |
| Strong Enhancer | 9 |
| Weak Enhancer | 8 |
| Repressed | 7 |
| Insulator | 6 |
| Repetitive/CNV | 5 |
| Transcription Transition | 4 |
| Transcription Elongation | 3 |
| Weak Transcription | 2 |
| Heterochromatin | 1 |

Rankings of each of the 10 chromatin state classes defined by the regression analysis described in Supplementary Table 2. The 10 classes (Column 1) and were ranked based on β coefficients from multivariable regression analysis on the full training and validation dataset (Column 2).
