# Supplementary material for: SuRFing the genomics wave: an R package for prioritising SNPs by functionality
Source: Genome Med. 2014 Oct 14;6(10):79. doi: 10.1186/s13073-014-0079-1 (PMC4224693; doi:10.1186/s13073-014-0079-1)
Supplement: Additional file 4: Table S4. — Position category rankings. [file 13073_2014_79_MOESM4_ESM.doc]

**Additional file Table S4: Position category rankings**

| Position | Rank |
| --- | --- |
| exon, splice site | 5 |
| promoter | 4 |
| 10 kb upstream and downstream of genes | 3 |
| CpG islands and CpG shores | 2 |
| intron | 1 |
| intergenic | 0 |

Rank orders of position categories, based on enrichment data presented by Schork and Hindorff.
