# Supplementary material for: SuRFing the genomics wave: an R package for prioritising SNPs by functionality
Source: Genome Med. 2014 Oct 14;6(10):79. doi: 10.1186/s13073-014-0079-1 (PMC4224693; doi:10.1186/s13073-014-0079-1)
Supplement: Additional file 5: Table S5. — Grid search parameter boundaries. [file 13073_2014_79_MOESM5_ESM.doc]

**Additional file Table S5: Grid search parameter boundaries**

| Model | MAF | Conservation | Chromatin States | DNase HS | Position | DNase Footprints | Enhancers | TFBSs |
| --- | --- | --- | --- | --- | --- | --- | --- | --- |
| ALL | 0-2 | 0-3 | 0-8 | 0-1 | 0-16 | 0-3 | 0-1 | 0-5 |
| DM | 0-13 | 0-7 | 0-8 | 0-1 | 0-18 | 0-2 | 0-1 | 0-6 |
| DFP | 0-1 | 0-1 | 0-6 | 0-1 | 0-15 | 0-6 | 0-6 | 0-3 |

For each of the three models ALL, DM and DFP (column 1), the table shows the
parameter boundaries of the grid search algorithm for each of the eight parameters listed in columns 2 to 9.
