# Supplementary figures and images for: SuRFing the genomics wave: an R package for prioritising SNPs by functionality
Source: Genome Med. 2014 Oct 14;6(10):79. doi: 10.1186/s13073-014-0079-1 (PMC4224693; doi:10.1186/s13073-014-0079-1)

# ClinVAR non-coding vs. matched 1KG background variants

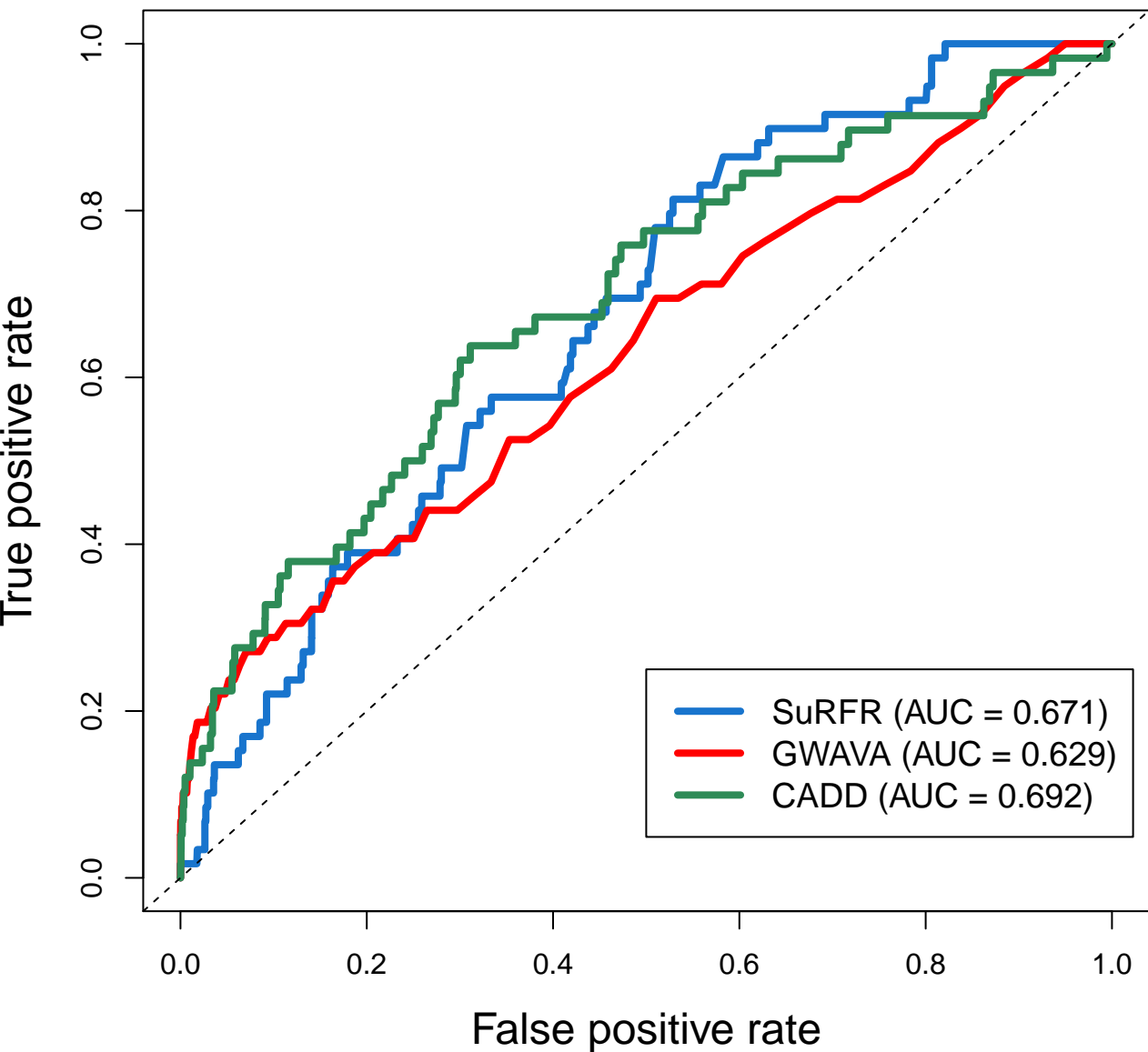

Supplement: Additional file 6: Figure S1. — Comparison of SuRFR, GWAVA and CADD on an additional, non-coding ClinVar dataset. ROC curves (true positive rate versus false positive rate) and AUCs for SuRFR, GWAVA and CADD run on a non-exonic, non-coding dataset of ClinVar pathogenic variants versus a matched 1000 Genomes background variant set. SuRFR, GWAVA and CADD perform to a similar level on these data. [file 13073_2014_79_MOESM6_ESM.pdf]

# HBB coding SuRFR vs GWAVA

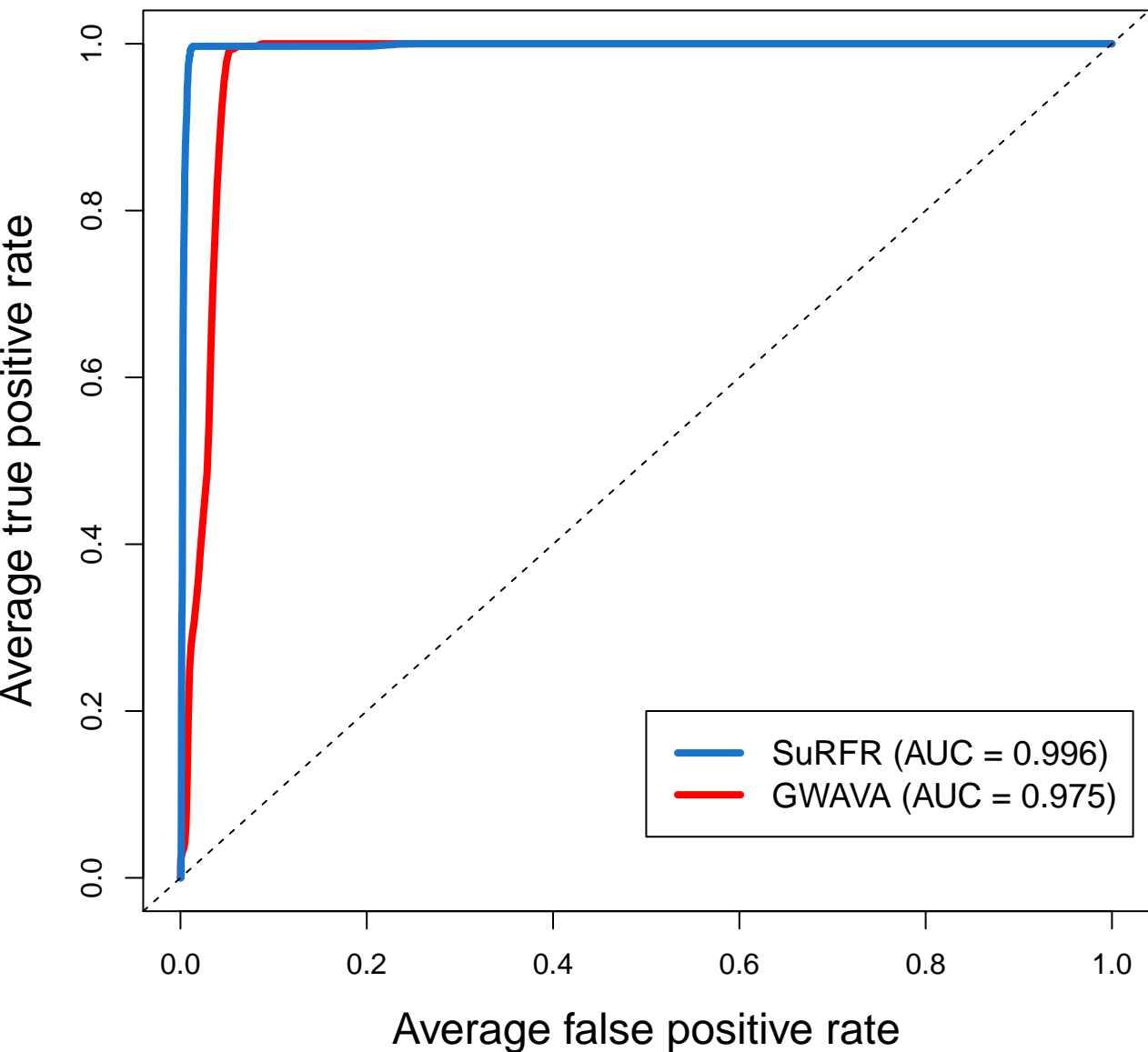

Supplement: Additional file 7: Figure S2. — ROC curves and AUCs of SuRFR versus GWAVA on HBB coding variants. The plot shows the performance of SuRFR and GWAVA in terms of true positive rates (x-axis) and false positive rates (y-axis), plotting ROC curves (SuRFR, blue; GWAVA, red) against performance expected by chance (grey dotted line). This figure shows that both methods are very good at prioritising functional coding variants over background variants. [file 13073_2014_79_MOESM7_ESM.pdf]

# RAVEN vs. matched background variants

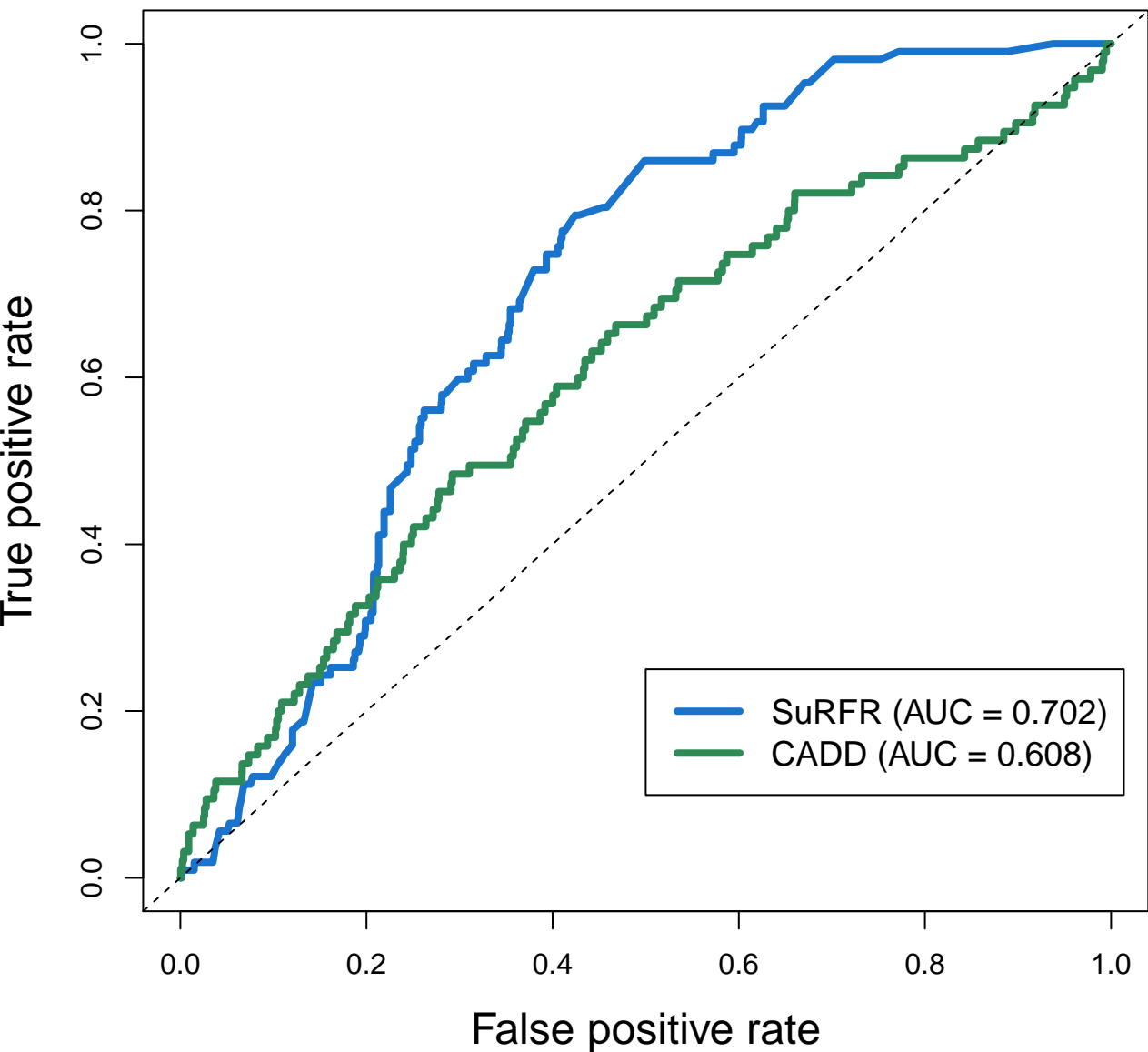

Supplement: Additional file 8: Figure S3. — ROC curves and AUCs for SuRFR versus CADD on RAVEN regulatory variants versus a matched control set. The plot shows the performance of SuRFR and CADD in terms of true positive rates (x-axis) and false positive rates (y-axis), plotting ROC curves (SuRFR, blue; CADD, green) against performance expected by chance (grey dotted line). This figure shows that both methods prioritise functional regulatory variants over matched background variants to a similar extent. [file 13073_2014_79_MOESM8_ESM.pdf]
